# Health protection among own account and platform workers during COVID19 in Chile. The PWR project: Marisol Ruiz

**DOI:** 10.1093/eurpub/ckac131.280

**Published:** 2022-10-25

**Authors:** M Ruiz, A Vives, V Alvarez, I Diaz, A Zaupa

**Affiliations:** 1 Department of Public Health, Pontificia Universidad Católica de Chile, Santiago, Chile; 2 Institute of Public Health, Universidad Austral de Chile, Valdivia, Chile; 3 CEDEUS, Pontificia Universidad Catolica, Santiago, Chile; 4 Sociology, University of Valparaiso, Valparaiso, Chile

## Abstract

**Background:**

In the last decades, non-standard employment has grown globally. Especially for own account workers (OAW), this implies the self-management of economic, health and other work-related risks. In the context of COVID19, this management was stressed by lockdowns and the novel health risks imposed by an unknown and highly contagious virus, demanding the incorporation of new knowledge and preventive actions. As part of a six-country multiple case qualitative study on non-standard workers (NSW), we explore their experiences and strategies deployed to protect their health while continuing to work.

**Methods:**

We performed 40 in-depth interviews to NSWs between October 2020 and February 2021, identified through the PWR online-survey and selected through an intentional sampling strategy according to levels of precarity (high-low), gender (male/female) and age (18-39/40-55). Interviews were analysed through abductive thematic analysis.

**Results:**

We observed a significant transfer to platform, Uber-like jobs in the delivery of goods during lockdown (n = 7). In the absence of institutional prevention programs and provision of protective equipment, OAWs (n = 13) refer the self-provision of COVID19 prevention to protect themselves and their families while continuing to work, deploying a series of strategies amidst limited understanding of both mode of transmission of the virus and actual effective preventive measures. This had serious consequences for them and their families, expressed in anguish, sense of lack of control, fear, and fragility in the face of a major health risk given their constant potential exposure to the virus, leading to both physical and mental health problems, as well as COVID19 infection.

**Conclusions:**

The substantial growth worldwide of gig delivery work during lockdowns magnified a pressing public health problem, critically requiring social security for gig and OAWs and the development of more equitable and accessible occupational health for all.

**Key messages:**

• Substantial growth worldwide of gig delivery work during lockdowns magnified a pressing public health problem.

• Social security for gig and OAWs are critically required as well as the development of more equitable and accessible occupational health for all.

